# MicroRNA-1908 functions as a glioblastoma oncogene by suppressing PTEN tumor suppressor pathway

**DOI:** 10.1186/s12943-015-0423-0

**Published:** 2015-08-12

**Authors:** Xuewei Xia, Yong Li, Wenbo Wang, Fang Tang, Jie Tan, Liyuan Sun, Qinghua Li, Li Sun, Bo Tang, Songqing He

**Affiliations:** Department of Neurosurgery, Guilin Medical University, Affiliated Hospital, Guilin, 541001 Guangxi People’s Republic of China; Department of Hepatobiliary Surgery, Guilin Medical University, Affiliated Hospital, Guilin, 541001 Guangxi People’s Republic of China; Laboratory of Liver Injury and Repair Molecular Medicine, Guilin Medical University, Guilin, 541001 Guangxi People’s Republic of China; Laboratory of Medical neurobiology, Guilin Medical University, Guilin, 541001 Guangxi People’s Republic of China; Department of Pathology and Physiopathology, Guilin Medical University, Guilin, 541004 Guangxi People’s Republic of China

## Abstract

**Background:**

We aimed to investigate whether miRNA-1908 is an oncogene in human glioblastoma and find the possible mechanism of miR-1908.

**Methods:**

We investigated the growth potentials of miRNA-1908-overexpressing SW-1783 cells *in vitro* and *in vivo*. In order to identify the target molecule of miRNA-1908, a luciferase reporter assay was performed, and the corresponding downstream signaling pathway was examined using immunohistochemistry of human glioblastoma tissues. We also investigated the miRNA-1908 expression in 34 patients according to the postoperative risk of recurrence.

**Results:**

The overexpression of miRNA-1908 significantly promoted anchorage-independent growth *in vitro* and significantly increased the tumor forming potential *in vivo*. MiRNA-1908 significantly suppressed the luciferase activity of mRNA combined with the PTEN 3’-UTR. Furthermore, the expression levels of miRNA-1908 were significantly increased in the patients with a high risk of recurrence compared to that observed in the low-risk patients, and this higher expression correlated with a poor survival.

**Conclusions:**

miRNA-1908 functions as an oncogene in glioblastoma by repressing the PTEN pathway. MiR-1908 is a potential new molecular marker for predicting the risk of recurrence and prognosis of glioblastoma.

**Electronic supplementary material:**

The online version of this article (doi:10.1186/s12943-015-0423-0) contains supplementary material, which is available to authorized users.

## Background

Glioblastomas, the prognosis of which is highly dependent on the histological grade, are the most common malignancies of the central nervous system in humans. New molecular targets and treatment strategies are urgently needed to combat this disease. MicroRNAs (miRNAs) are small, endogenous noncoding RNAs composed of 18–23 nucleotides (nt) that post-transcriptionally regulate gene expression by targeting the 3’-untranslated regions of mRNAs [1–3]. Many miRNAs are proto-oncogenes or tumor suppressors [4–6], and their functions have been extensively studied in various cancer types, including glioblastoma [7–10]. Recent studies using genome-wide approaches have revealed that miRNAs, such as miR-7 [11], miR-128 [12], and miR-21 [13], are globally dysregulated in glioblastoma. Of note, there are some microRNAs differently expressed in adult glioblastomas and paediatric glioblastoma [14]. Our aim was to investigate the role and mechanism of microRNAs in glioblastomas that could contribute to the diagnosis and prognostic evaluation of glioblastoma patients. MiR-1908 is a novel microRNA that is highly expressed in human adipocytes [15]. But the potential role of miR-1908 in the carcinogenesis and tumor development of glioblastoma is unknown.

Indeed, mounting evidence has shown that the poor prognosis of patients with glioblastoma and therapeutic failure are associated with a number of abnormally activated signaling pathways, among which phosphoinositide 3-kinase (PI3K)/AKT signaling represents one of the most important regulatory pathways for the malignancy [16, 17]. Notably, aberrant Akt activation is a poor prognostic factor for glioblastoma of all stages and contributes to resistance to first-generation single-agent targeting therapy such as gefitinib, a tyrosine kinase inhibitor clinically used for patients with glioblastoma with EGFR over-activation [18, 19]. Biologically, activated AKT confers glioblastoma cells resistant to chemotherapy and radiation and promotes cancer cell survival, and in contrast, chemically synthetic compounds inhibiting AKT activation induce apoptosis of glioblastoma cells *in vitro* as well as *in vivo* [20]. Moreover, AKT signaling contributes to oncogenesis through activating multiple downstream effector molecules. Of note, activated AKT phosphorylates tumor suppressor FOXO3a and impairs the transcription of its target genes related to cell growth arrest such as p21, inactivation of which has also been implicated in the promotion of tumor angiogenesis [21, 22]. In addition, mTOR, another substrate subjected to phosphorylation by AKT, enhances phosphorylation of S6K1 and 4E-BP1 [23] and plays crucial roles in the regulation of ribosomal protein synthesis, for example, production of cyclin D1 and VEGF-A at both transcriptional and translational levels [24, 25]. It has been found that mediated by the above molecular mechanisms, both AKT/FOXO3a and AKT/mTOR pathways underlie lung cancer development and progression [26, 27]. Thus, inhibitors targeting these pathways might represent potentially applicable therapeutic agents against glioblastoma.

In the current study, we identify that miR-1908 is highly expressed in multiple subtypes of glioblastoma tissues and causes simultaneous downregulation of PTEN, leading to activation of both AKT/FOXO3a and AKT/mTOR pathways, consequently leading to accelerated proliferation and enhanced angiogenesis in glioblastoma.

## Results

### Aberrant expression of miR-1908 in human glioblastoma cells was correlated with poor prognosis

**Fig. 1:**
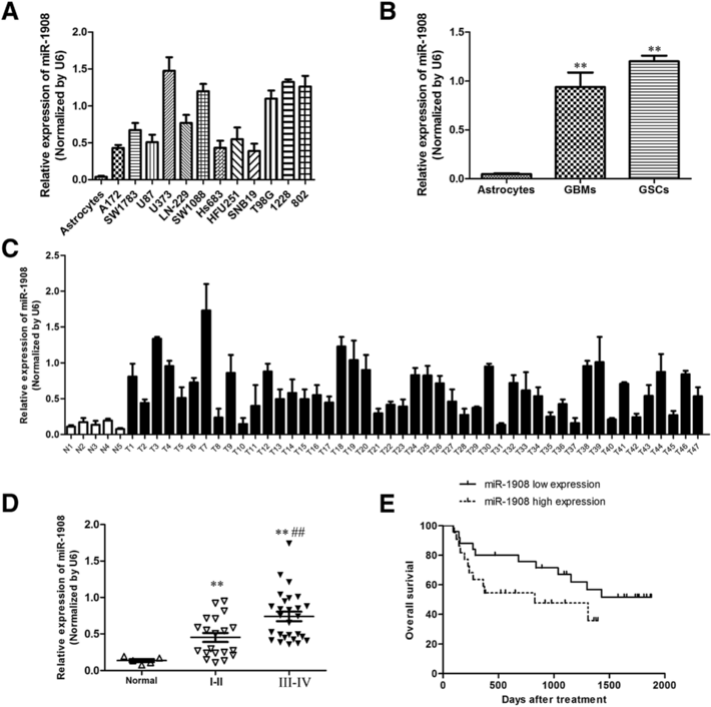
miR-1908 is upregulated in glioblastoma cells, GSCs, and human tumors and inversely correlates with patient survival. **a** Quantification of miR-1908 in glioblastoma cell lines (A127, SW1783, U87, U373, LN-229, SW1088, HS683, HFU251, SNB19 and T98G)) and stem cell lines (GSCs; 802 and 1228) showing higher expression than in normal human astrocytes. **b** Average expression of miR-1908 in glioblastoma cells, GSGs and normal human astrocytes. **c** Quantification of miR-1908 in human glioblastoma tumors (T) and normal human brain (N). **d** Quantification of miR-1908 in normal brain, stageI-IIglioblastoma and stage III-IV glioblastoma, (**e**) Correlation analysis of expression data and patient survival data from Affiliated Hospital of Guilin Medical University showing that miR-1908 levels are a risk indicator for survival. **p < 0.01 and ##*p* < 0.01 based on the Student *t* test. ##: Student *t* test between stage I-IIglioblastoma and stage III-IV glioblastoma. Error bars, SD

We first measured miR-1908 levels in glioblastoma cells (A127, SW1783, U87, U373, LN-229, SW1088, Hs683, HFU251, SNB19, and T98G). As shown in Fig. 1a, miR-1908 was significantly higher in glioblastoma cells (Fig. 1a) than in astrocytes. Moreover,miR-1908 was expressed higher in both glioblastoma (GBMs) and glioblastoma stem cells (GSCs) (Fig. 1b) than that in astrocytes. These results indicated that miR-1908 may be related to the growth and recurrence of glioblastomas. To further confirm that miR-1908 is related with the development of glioblastoma, we measured the miR-1908 expressions in 47 glioblastoma samples (Table 1). As shown in Fig. 1c, miR-1908 was significantly higher in glioblastoma than in normal brain (Fig. 1c). Of note, miR-1908 was highest in stageIII-IV tumors and higher in stageI-II tumors than in normal brain (Fig. 1d) showing us that miR-1908 may be a prognostic factor of glioblastoma.

**Table 1:** Clinicopathological features of 47 patients with gliomas

Features	WHO I	WHO II	WHO III	WHO IV
NO. of patients	12	8	15	12
Mean age (years)	46.8	53.6	49.5	56.3
Gender				
Male	7	6	12	7
Female	5	2	3	5
KPS				
≥80	8	7	4	3
<80	4	1	11	9
Surgery				
Gross total resection	12	8	9	6
Partial resection	0	0	5	4
Biopsy	0	0	1	2
Adjuvant treatment				
Radiotherapy	0	0	13	8
Chemotherapy	0	0	0	1
Radiotherapy and chemotherapy	0	0	2	3

*KPS* karnofsky Performance Scale score, *WHO* World Health Organization grade

To further evaluate whether miR-1908 is related with prognosis of glioblastoma patients, we carried out bioinformatics analysis. In survival analysis of glioblastoma patients, we found that patients with higher miR-1908 expression levels had poorer disease free survival (DFS) than those with lower miR-1908 expression levels (Fig. 1e) which suggested that miR-1908 significantly affected prognosis of glioblastoma patients. Altogether, these data demonstrate that miR-1908 is upregulated in glioblastoma and that high miR-1908 expression predicts poor patient survival.

### The effects of miR-1908 on proliferation of glioblastoma

**Fig. 2:**
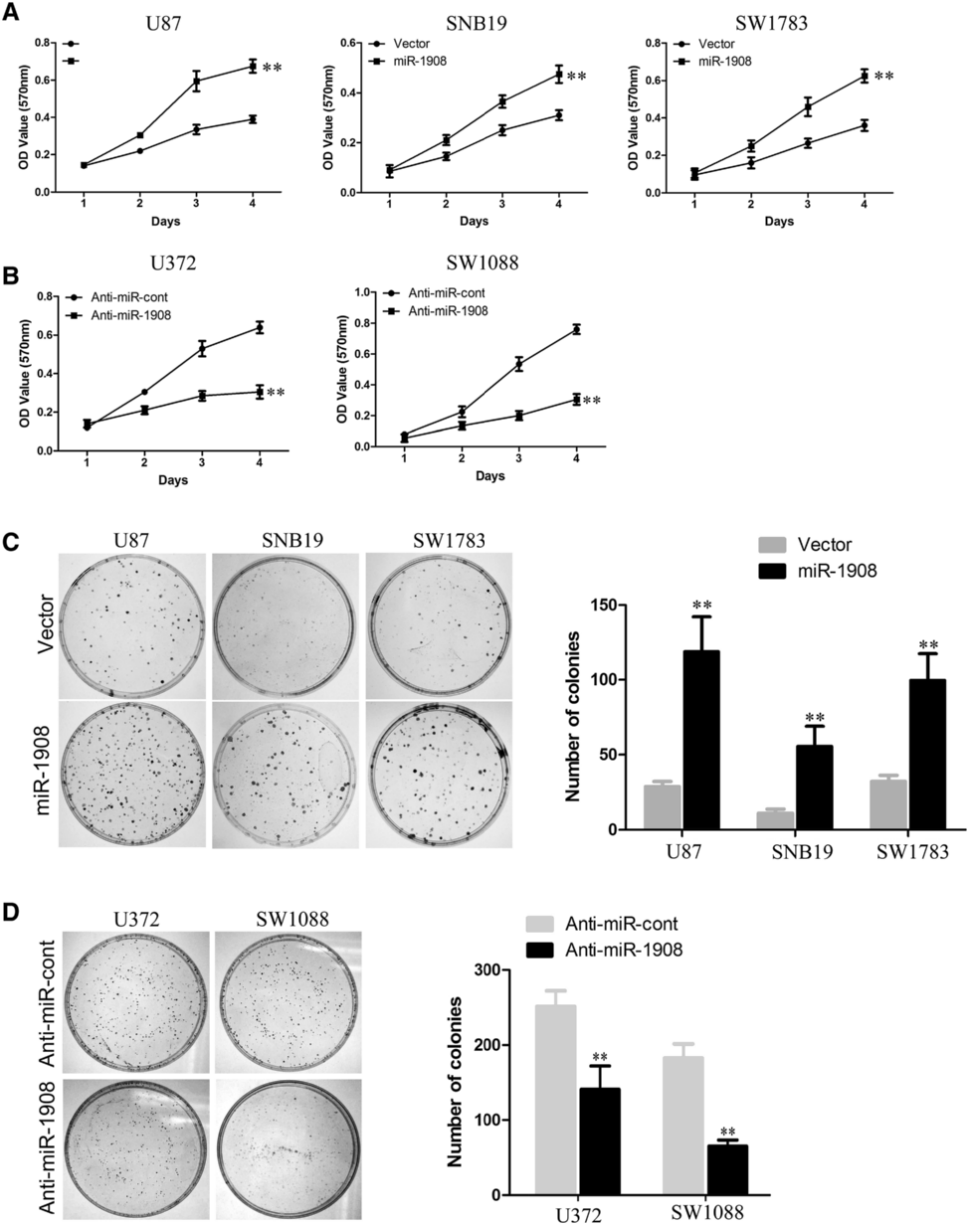
Ectopic miR-1908 expression in glioblastoma cells accelerates proliferation of glioblastoma cells. **a** MTT assay reveals cell growth curves of SNB19, U87, SW1783 cells. **b** MTT assay reveals cell growth curves of U373, SW1088 cells. **c** Representative micrographs of crystal violet-stained cell colonies analyzed by colony formation assay for 14 days. **d** Representative micrographs of crystal violet-stained cell colonies analyzed by colony formation assay for 14 days. **P < 0.01 based on the Student *t* test. Error bars, SD

To better stand the role of miR-1908 in glioblastoma, we used retroviral vectors to establish glioblastoma cell lines stably overexpressing or silencing miR-1908. The expression levels of miR-1908 in the subsequent cell lines were examined by qRT-PCR (Additional file 1: Figure S1 A-E). Firstly we used 3-(4,5-dimethylthiazol-2-yl)-2,5-diphenyltetrazolium bromide (MTT) and colony formation assays to investigate a growth-promoting effect of miR-1908 on glioblastoma cells. MTT assay revealed that overexpression of miR-1908 promoted proliferation of glioblastoma cells (Fig. 2a). In colony formation assay, overexpression of miR-1908 significantly increased the viability of indicated cells which formed more and bigger clones (Fig. 2c). In contrast, silencing miR-1908 in glioblastomas dramatically suppressed the proliferation (Fig. 2b) and viability (Fig. 2d) of indicated cells.

**Fig. 3:**
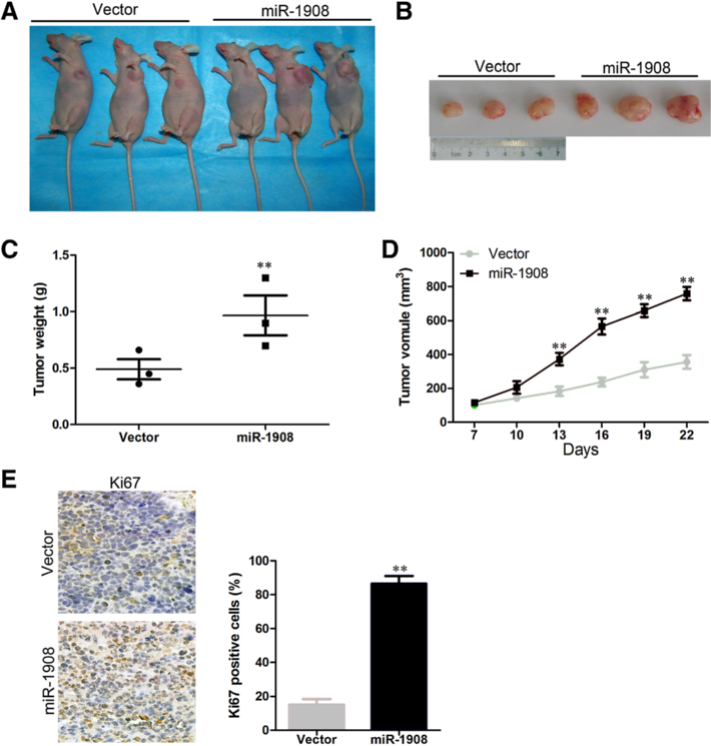
miR-1908 suppresses glioblastoma cells tumor growth *in vivo*. **a** U87 cells stably overexpressing miR-1908 or scrambled miRNA was subcutaneously injected into nude mice. 22 days later, U87 cells stably overexpressing miR-1908 had bigger tumors than controls. **b** Representative picture of tumors formed. **c** Tumor weight. Data is presented as the mean ± SD. **d** Growth curves of tumor volumes. **e** Hematoxylin and eosin (H&E) staining confirms tumor cells in slices of indicated tumor sections immunohistochemically stained for Ki-67. **P < 0.01 based on the Student *t* test. Error bars, SD

In order to confirm whether the growth-promoting effect of miR-1908 observed in cultured cells is relevant to glioblastoma growth *in vivo*, U87-miR-1908 cell and control cell were subcutaneously inoculated into BALB/C athymic mice, respectively. As show in Fig. 3a and b, overexpression of miR-1908 significantly accelerated tumor growth and induced an increase in tumor weight (Fig. 3c) and volume (Fig. 3d). As Ki-67 indicates the proliferative ability of tumor, we examined Ki-67 in xenograft tumor sections. As shown in Fig. 3e, higher Ki-67 levels were found in sections with overexpressing miR-1908 by ICH. Taken all together, we concluded that UBE2T promote the proliferation of prostate cancer cells *in vivo* and *in vitro*.

### MiR-1908 promotes invasion and sphere formation in glioblastoma cells

**Fig. 4:**
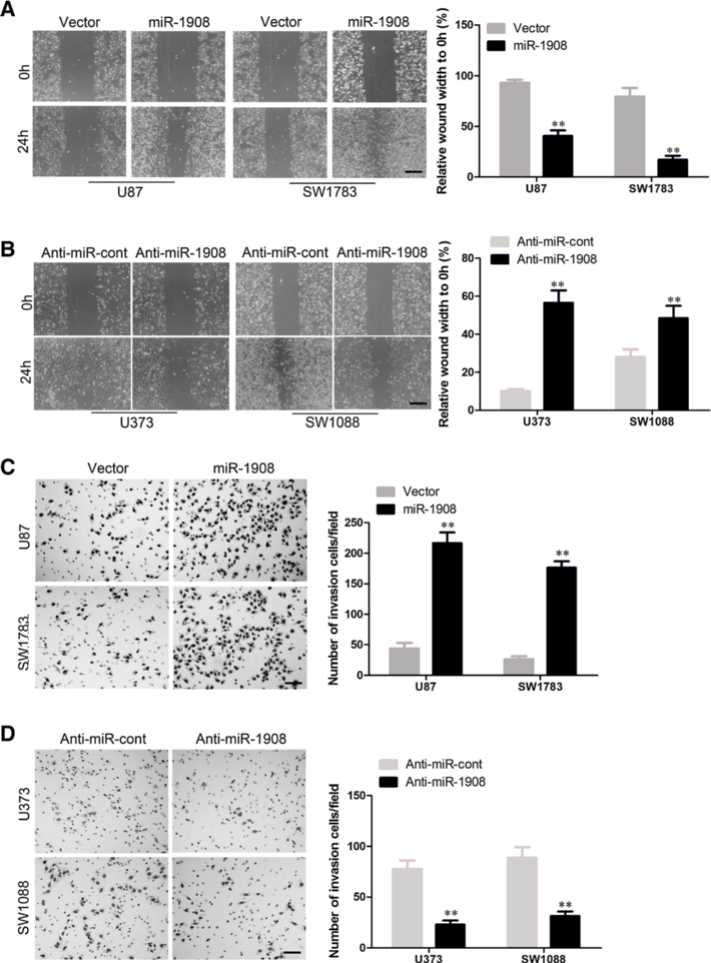
Overexpressing miR-1908 promotes migration and invasion of glioblastoma cells. **a** Glioblastoma cell lines were transfected with miR-1908 and assessed for migration with the wound-healing assay. **b** Glioblastoma cell lines were transfected with anti-miR-1908 and assessed for migration with the wound-healing assay. **c** Representative results of invasive ability of U87 and SW1783 cells transfected with miR-1908 mimics or miR control. **d** Representative results of invasive ability of U373 and SW1088 cells transfected with anti-miR-1908 mimics or miR control. **P < 0.01 based on the Student *t* test. Error bars, SD

To further understand the function of miR-1908 in glioblastoma, we next assessed the effect of miR-1908 on glioblastoma cell migration and invasion. In scratch assay, overexpression of miR-1908 significantly accelerated wound healing of glioblastoma cells (Fig. 4a and Additional file 2: Figure S2A) while silencing miR-1908 decreased the rate of migration (Fig. 4b). Moreover, matrigel assay was used to evaluate the invasive ability of glioblastoma, in results, ectopic expression of miR-1908 significantly enhanced the invaded rate of glioblastoma cells (Fig. 4c and Additional file 2: Figure S2B)) meanwhile silencing miR-1908 decreased the number of invaded cells (Fig. 4d).

**Fig. 5:**
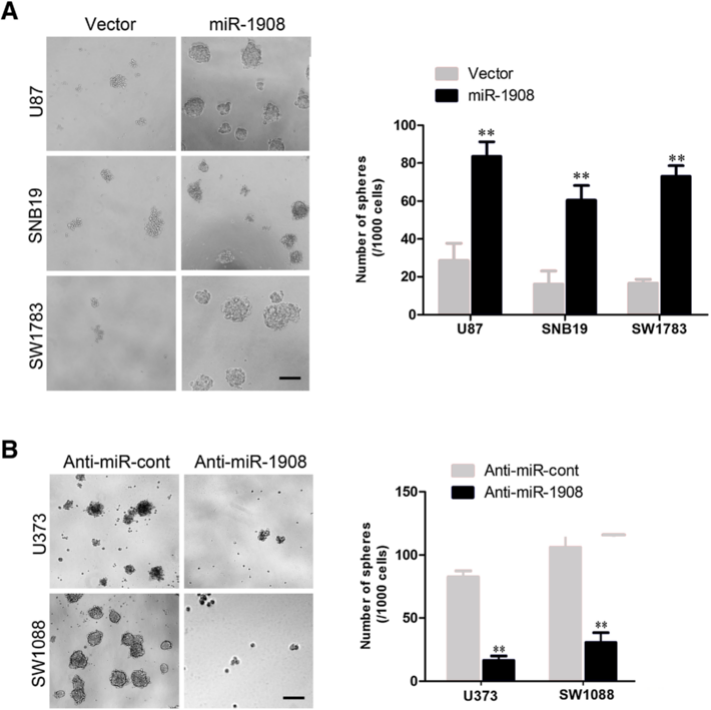
Ectopic miR-1908 expression in glioblastoma cells accelerates sphere formation. **a** Representative results of sphere formation of SNB19, U87 and SW1783 cells transfected with miR-1908 mimics or miR control. **b** Representative results of sphere formation of SW1088, U373 cells transfected with anti-miR-1908 mimics or miR control. **P < 0.01 based on the Student *t* test. Error bars, SD

We have confirmed that miR-1908 was expressed higher in GSCs (Fig. 1b). To further confirm the role of miR-1908 in GSCs, sphere formation was used to reveal the function of miR-1908 in GSCs. As shown in Fig. 5a, overexpression of miR-1908 promoted the formation of spheres of glioblastoma cells (Fig. 5a) meanwhile silencing miR-1908 decreased the number of spheres (Fig. 5b).

### PTEN is a potential target of miR-1908, and PTEN levels are inversely correlated with miR-1908 levels in glioblastoma tissues

**Fig. 6:**
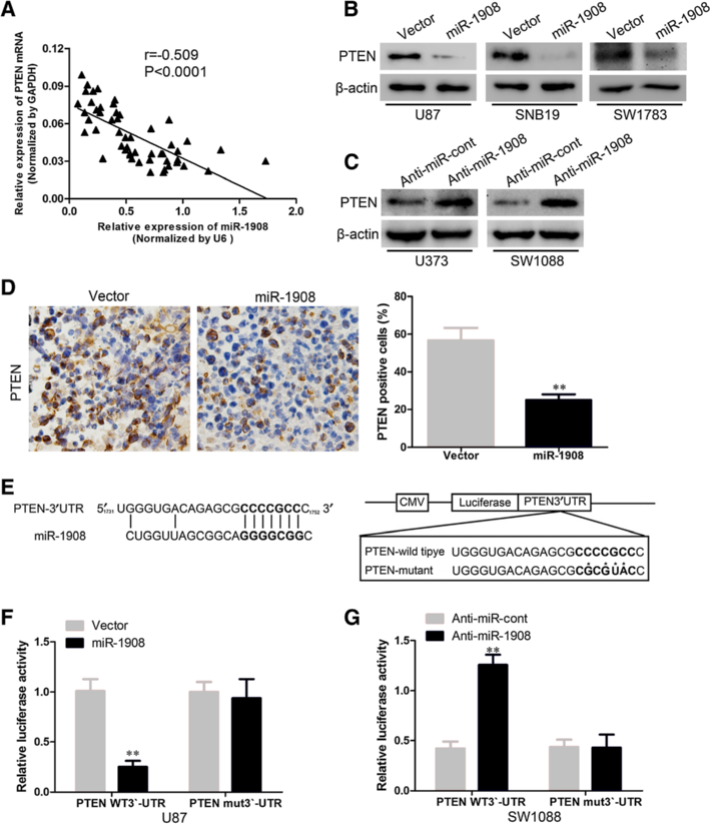
PTEN is a direct target of miR-1908. **a** Correlation of miR-1908 overexpression with PTEN downregulation in indicated glioblastoma tissues. **b** PTEN expression in miR-1908 overexpressing cells in western blot. **c** PTEN expression in miR-1908 silencing cells in western blot. **d** ICH for PTEN in miR-1908 overexpressing tumors and control. **e** Wild-type miR-1908 target sequences of *PTEN* 3’-UTR and mutant-type miR-1908 target sequences of *PTEN* 3’-UTR. **f** and **g** Relative luciferase activity of PTEN in cells after co-transfection with wild type (Wt) or mutant (Mt) *PTEN* 3’-UTR reporter genes and miR-1908 mimics, anti-miR-1908 mimics or control. **P < 0.01 based on the Student *t* test. Error bars, SD

To verify whether PTEN is a potential target of miR-1908, we analyzed the relation between miR-1908 and PTEN. In linear correlation analysis, PTEN expression was inversely proportional to miR-1908 expression (Fig. 6a). To confirm this inverse relationship, we verified PTEN levels in glioblastoma cells. As shown in Fig. 6b, overexpressing miR-1908 significantly suppressed PTEN levels in glioblastoma cells (Fig. 6b) meanwhile silencing miR-1908 increased PTEN expression (Fig. 6c). Moreover, PTEN was significantly suppressed in xenograft tumor sections with overexpression of miR-1908 (Fig. 6d). To further confirm the regulation of PTEN by miR-1908, the luciferase reporter containing the complimentary seed sequence of miR-1908 at the 3’-UTR region of PTEN mRNA was constructed (Fig. 6e), luciferase activity was detected at 48 h after the co-transfection of FLuci vector (3-UTR-PTEN wt FLuci vector or 3 -UTR-PTEN mut FLuci vector), miR-1908 mimic or NC mimic, and RLuci vector in glioblastoma cells. As shown in Fig. 6f and g and Additional file 3: Figure S3, the luciferase activity was significantly decreased in glioblastoma cells co-transfected with 3’-UTR-PTENwt FLuci vector and miR-1908 mimic compared with those co-transfected with 3’-UTR-PTENmut FLuci vector and miR-1908 mimic, suggesting that the fragment at the 3’-UTR of the PTEN mRNA was the complementary site for the miR-1908 seed region (Fig. 6f, Additional file 3: Figure S3A and B), meanwhile silencing miR-1908 increased the luciferase activity of PTEN (Fig. 6g, Additional file 3: Figure S3C). These results revealed that miR-1908 directly mediates the degradation of PTEN mRNA.

### Both AKT/FOXO3a and AKT/mTOR signaling contribute to miR-1908-mediated malignant phenotype of glioblastoma cells

**Fig. 7:**
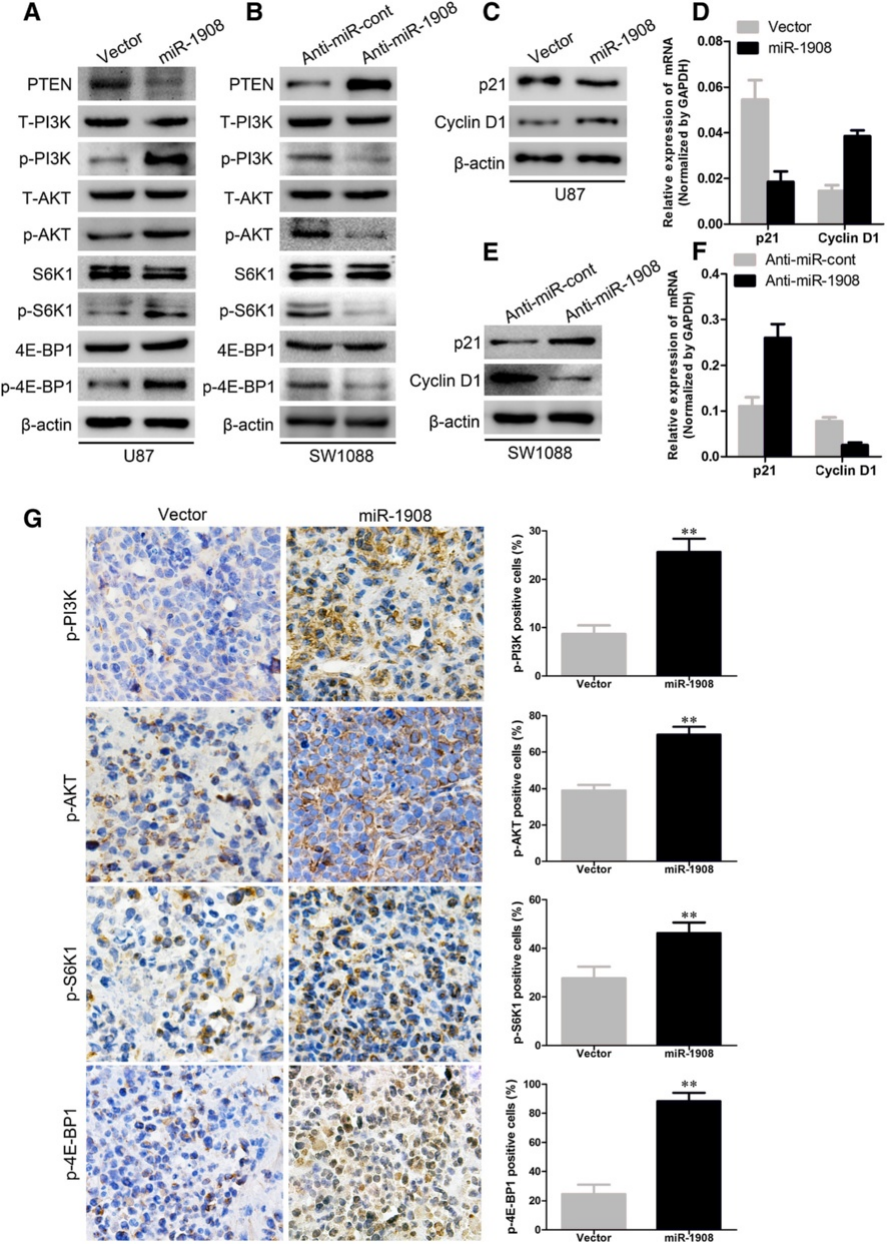
miR-1908 activates both AKT/FOXO3a and AKT/mTOR signaling pathway. **a** and **b** Western blot analysis of phospho-P13K, total P13K (T-AKT), phospho-AKT, total AKT (T-AKT), phospho-S6K1, total S6K1, phospho-4E-BP1, and total 4E-BP1 in indicated cells. **c, d, e** and **f** Protein expression and relative mRNA quantitation of p21 and cyclin D1 in indicated cells. **g** ICH for phosphor-P13K, phospho-AKT, phospho-S6K1 and phospho-4E-BP1 in miR-1908 overexpressing tumors. **P < 0.01 based on the Student *t* test. Error bars, SD

**Fig. 8:**
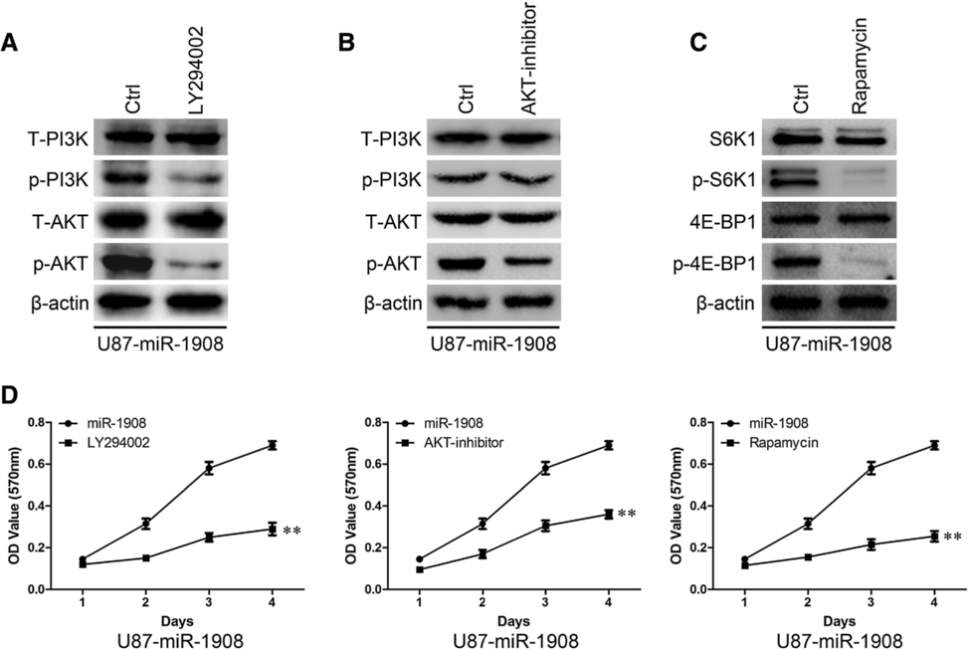
AKT/FOXO3a or AKT/mTOR signaling pathway inhibitors restrained the proliferation of miR-1908 overexpressing cells. **a, b** and **c** Western blot analysis shows the effect of LY294002, AKT inhibitor III or rapamycin. **d** MTT shows the effect of LY294002, AKT inhibitor III or rapamycin on miR-1908 overexpressing cells. **P < 0.01 based on the Student *t* test. Error bars, SD

We next examined the role of miR-1908–mediated inhibition of PTEN in the development and maintenance of the malignant phenotype of glioblastoma cells. Of note, ectopic miR- 1908 expression in U87 cell remarkably increased the level of phosphorylated AKT, resulting in enhanced phosphorylation of P13K, S6K, and 4E-BP1 (Fig. 7a), whereas silencing miR-1908 in SW1088 cells robustly suppressed phosphorylation of AKT, P13K, S6K, and 4E-BP1 (Fig. 7b), indicating that miR-1908 indeed activated the AKT/FOXO3a and AKT/mTOR pathways. To confirm the activation of AKT/FOXO3a and AKT/mTOR pathways, we next examined the levels of P21 and Cyclin D1 which were the targets of AKT pathway. As shown in results, decreased P21 and increased Cyclin D1 proteins (Fig. 7c) and mRNA (Fig. 7d) could be caused by miR-1908 overexpression, whereas opposite effects on the regulation of P21 and Cyclin D1 were found at both the mRNA and protein levels when miR-1908 was knocked down (Fig. 7e and f). Moreover, increased phosphorylation of P13K, S6K, and 4E-BP1 were found in tumors overexpressing miR-1908. To further confirm the activation of AKT/FOXO3a and AKT/mTOR pathways, we used different inhibitors to inhibit the pathways. As shown in results, P13K inhibitor LY294002 significantly decreased phosphorylation of P13K and Akt (Fig. 8a); Akt inhibitor decreased phosphorylation of Akt but not P13K (Fig. 8b); mTOR inhibitor rapamycin decreased phosphorylation of S6K and 4E-BP1 (Fig. 8c). And all these three inhibitors suppressed the proliferation of miR-1908 overexpressing U87 cells (Fig. 8d). These results suggested that miR-1908 promoted the proliferative and angiogenic phenotype of glioblastoma cells by simultaneously activating both AKT/FOXO3a and AKT/mTOR pathways.

### Repression of PTEN in glioblastoma cells was essential for miR-1908-induced proliferation

**Fig. 9:**
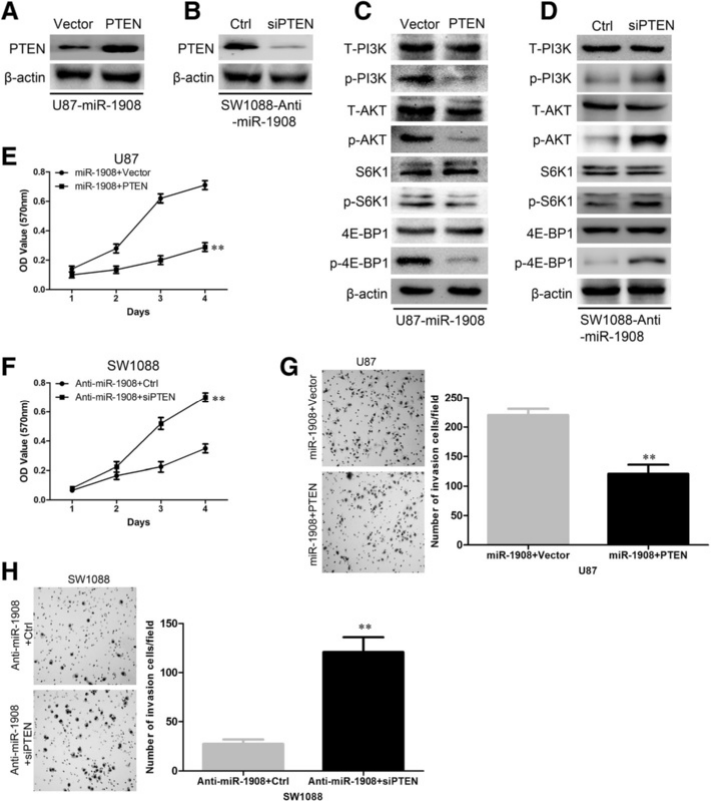
Restoration of PTEN inverses miR-1908–induced proliferation and invasion. **a** and **b** Western blotting confirmation of reexpression of PTEN, as well as depletion of PTEN in indicated cells. **c** and **d** Western blot analysis of indicated proteins in indicated cells. **e** and **f** MTT shows the effect on indicated cells after reexpression or depletion of PTEN. **g** and **h** Matrigel assay shows the effect on indicated cells after reexpression or depletion of PTEN. **P < 0.01 based on the Student *t* test. Error bars, SD

On the basis of the indispensable role of PTEN in the biologic functions of miR-1908, we overexpressed PTEN in U87-miR-1908 cell (Fig. 9a) and silenced PTEN in SW1088-anti-miR1908 cell (Fig. 9b). As shown in Fig. 9c and d, PTEN overexpression significantly inhibited phosphorylation of Akt, P13K, S6K, and 4E-BP1 in U87-miR-1908 cells (Fig. 9c), meanwhile silencing PTEN increased phosphorylation of P13K, S6K, and 4E-BP1 in SW1088-anti-miR1908 cell (Fig. 9d). In functional tests, PTEN overexpression in U87-miR-1908 cell significantly decreased the proliferation in MTT assay (Fig. 9e) and colony formation assay (Additional file 4: Figure S4A) meanwhile silencing PTEN promoted the proliferation of SW1088-anti-miR1908 cells (Fig. 9f, Additional file 4: Figure S4B). Moreover PTEN overexpression significantly inhibited wound healing (Additional file 4: Figure S4C) and invasion ability (Fig. 9g) of U87-miR-1908 cells, meanwhile silencing PTEN promoted wound healing (Additional file 4: Figure S4D) and invasion ability (Fig. 9h) of SW1088-anti-miR1908 cells. These data suggested that PTEN wan essential for miR-1908-induced proliferation, migration and invasion.

## Discussion

Recently, miRNAs have been shown to be important in maintenance of normal cellular function, and the dysregulation of miRNAs expression can result in cancer initiation and tumor progression [28–30]. MiR-1908 is a new member of the microRNA family. In this study, we investigated the expression, function, and mechanism of miR-1908 in glioblastoma. We found that miR-1908 is a risk factor in glioblastoma where it acts as an oncogene by regulating PTEN expression. In glioblastoma cells, overexpression of miR-1908 robustly promotes cell proliferation and invasion *in vitro*. In contrast, inhibition of endogenous miR-1908 remarkably abrogates the proliferation and invasion of glioblastoma cells. At the molecular level, both the AKT/FOXO3a and AKT/mTOR pathways contribute to miR-1908–mediated malignant phenotype of glioblastoma cells, likely mediated by suppressing PTEN expression. Of note, the close correlation between high miR-1908 expression and low expression of PTEN, as well as with the malignant properties of glioblastoma tumors, were also confirmed in planted tumors and in clinical glioblastoma samples, suggesting a possible role of miR-1908 in the development and progression of glioblastoma.

Each miRNA has the potential to target hundreds of genes that harbor target sequence in their 3’-UTR complementary to the seed region of the miRNA [1]. PTEN is one of the most frequently mutated tumor suppressors in human cancer including brain tumors [31, 32]. PTEN loss was considered to be one of three oncogenic factors in glioblastomas [33]. PTEN also suppresses migration; genetic deletion of the *Pten* tumor suppressor gene promotes cell motility [34], and PTEN reconstitution or overexpression inhibits cell motility in a variety of cell types [35]. Mechanistically, PTEN reduces cell motility through a variety of pathways, and P13K/AKT is one important target of PTEN [36]. In this study, overexpression of miR-1908 significantly decreased PTEN in glioblastoma cells to inhibit phosphorylated P13K and AKT, resulting in increase in proliferation, migration and invasion [37–39]. PTEN overexpression could restrain the increase in proliferation, migration and invasion in miR-1908-overexpresison glioblastoma cells.

Clinical studies have revealed that PTEN mutation in glioblastoma has no correlation with survival [40]. Nevertheless, in anaplastic oligodendroglioblastomas and astrocytomas there was a positive correlation between PTEN alterations and poor prognosis [40, 41]. Furthermore, elevated AKT activity has been associated with poor prognosis [42]. Paediatric patients harbouring PTEN mutation in tumours have poorer prognosis [43]. Thorarinsdottir et al. reported that deficient PTEN expression was associated with worse overall survival in childhood high grade glioblastomas [44]. Our findings could provide new guidance for glioblastoma treatment and improve prognoses in the future.

## Materials and methods

### Clinical specimens and cell culture

A total of 47 giloma specimens and five normal brain samples frozen in liquid nitrogen were obtained from Affiliated Hospital of Guilin Medical University. No patients had received any anti-tumor treatments before biopsy. The human glioblastoma cell lines (A127, SW1783, U87, U373, LN-229, SW1088, Hs683, HFU251, SNB19, T98G, 1228 and 802) were cultured in RPMI-1640 (Invitrogen) supplemented with 10 % FBS (Gibco) and 1 % streptomycin/penicillin at 37 °C with 5 % CO_2_.

### RNA extraction, reverse transcription, and real-time RT-PCR

Total RNA was extracted from freshly-frozen samples or cells with TRIzol reagent (Invitrogen). Total RNA was reverse-transcribed with First Strand cDNA Synthesis Kit (Invitrogen). Real time PCR reactions were conducted using Platinum SYBR Green qPCR SuperMix-UDG reagents (Invitrogen) on the PRISM 7900HT system (Applied Biosystems). All reactions were done in triplicate and reactions without reverse transcriptase were used as negative controls. The U6 or GAPDH were used as the endogenous controls and the 2-^ΔΔCT^ equation was used to calculate the relative expression levels.

### Oligonucleotide transfection and generation of stably transfected cell lines

Cells were seeded into 6-well plates, transfected with miR-1908 mimics or miR controls (50 nM, GenePharma) using Lipofactamine™ RNAiMAX (Invitrogen) and transfected with siMIF (100 nM, Invitrogen) or siRNA controls using Lipofactamine 2000 reagent (Invitrogen), and then harvested for assays 48 h later. The lentiviral plasmid pEZX-MR03 (GeneCopoeia) expressing 1908 (Cat, HmiR0274-MR03) or scrambled miRNA (Cat, CmiR0001-MR03) and Lenti-Pac HIV Expression Packaging mix (GeneCopoeia) were cotransfected into glioblastoma cells using EndoFectin Lenti transfection reagent (GeneCopoeia). After transfection for 48 h, lentiviral particles were harvested and then transduced into the glioblastoma cells, and the stably transfected cells were selected using puromycin and validated by real time Western blot.

### MTT assay and colony formation assay

Glioblastoma cells were seeded at 1500 cells per well in 96-well plates after transfection. MTT assay was performed to test cell viability at 1, 2, 3, and 4 days, and the absorbance was measured at 490 nm with a spectrophotometric plate reader. For colony formation assay, cells were plated at 500 cells per well in six-well plates after transfection, and cultured for 14 days. Colonies were fixed with methanol, stained with 0.5 % crystal violet, and counted under the inverted microscope.

### Western blot analysis

Western blot analysis was conducted using anti-phospho-AKT (ser473), anti-AKT, anti-FOXO3a, anti-phospho-S6K1 (Thr389), anti-S6K1 and anti-4E-BP1(Epitomics), anti-phospho-FOXO3a (ser253), and phospho-4E-BP1 (Ser65; Cell Signaling Technology), anti-p21, anti-cyclinD1 and anti-PTEN (BD PharMingen) antibodies.

### Cell migration and invasion assays

The effects of miR-1908 or PTEN expression on cell migration and invasion were assessed using the wound-healing and Transwell assays as previously described [45].

### *In vivo* tumor growth model

Male BALB/c nude mice aged 4 to 6 weeks were purchased from the Hunan Slac Jingda Laboratory Animal Co., Ltd (Changsha, China). For tumor growth assay, SUNE-1 cells stably overexpression miR-451 or scramble miRNA were resuspended in PBS and 1 × 106 cells (200 μl) were subcutaneously injected in the dorsal flank of nude mice. Tumor size was measured every 3 days and tumor volumes were calculated with the following formula: volume = (L × W2)/2, in which L meant the longest diameter and W meant the shortest diameter. Four weeks later, mice were sacrificed, and tumors were dissected and weighted. Animal handling and research protocols were approved by the Animal Care and Use Ethnic Committee.

### Immunohistochemical analysis

After 19 days, mice were anesthetized and sacrificed, and tumors were removed, photographed, weighed, and sectioned (5 mm in thickness), followed by immunostaining. Following deparaffinization, sections were immunohistochemically analyzed using antibodies for miR-1908, Ki67, p-Akt, p-P13K, p-S6K1, and p-4E-BP1, respectively, and subsequently were pathologically confirmed for the tumor phenotype and specific immunostaining. The positive cells were counted and analyzed.

### Luciferase reporter assay

The 3’-UTR (untranslated region) sequence of PTEN was predicted to interact with miR-1908 or a mutated sequence within the predicted target sites was synthesized and inserted into the XbaI and FseI sites of the pGL3 control luciferase reporter vector (Promega, Madison, WI). The luciferase reporter assay was performed as previously described [46].

## Conclusions

MiR-1908 expression is frequently up-regulated in glioblastoma. Overexpression of miR-1908 promotes the malignant phenotype of glioblastoma cells by promoting cell proliferation, migration and invasion through silencing PTEN expression. These findings indicate that miR-1908 plays an important role as a tumor promotor in glioblastoma development.

## Additional files

Additional file 1: Figure S1. Confirmation of miR-1908 expression in indicated cells. **(A), (B), (C), (D) and (E)** Quantification of miR-1908 in indicated cells. ***P* < 0.01. based on the Student *t* test. Error bars, SD. (JPEG 2379 kb) Additional file 2: Figure S2. miR-1908 promotes migration and invasion in SNB19 cells. **(A)** Scratch assay shows the effect of miR-1908 on migration in SNB19 cells. (B) Matrigel assay shows the effect of miR-1908 on invasion in SNB19 cells. ***P* < 0.01 based on the Student *t* test. Error bars, SD. (JPEG 2042 kb) Additional file 3: Figure S3. miR-1908 inhibits PTEN activation in glioblastoma cells. **(A), (B) and (C)** Relative luciferase activity of PTEN in cells after co-transfection with wild type (Wt) or mutant (Mt) *PTEN* 3’-UTR reporter genes and miR-1908 mimics, anti-miR-1908 mimics or control. ***P* < 0.01 based on the Student *t* test. Error bars, SD. (JPEG 1115 kb) Additional file 4: Figure S4. Restoration of PTEN inverses miR-1908–induced proliferation and invasion in U87 or SW1088 cells. Colony formation **(A)** and scratch assay **(C)** show the effect on indicated cells after reexpression of PTEN. Colony formation **(B)** and scratch assay **(D)** show the effect on indicated cells after depletion of PTEN. ***P* < 0.01 based on the Student *t* test. Error bars, SD. (JPEG 4468 kb)

## References

[1] Bartel DP (2004). MicroRNAs: genomics, biogenesis, mechanism, and function. Cell.

[2] He L, Hannon GJ (2004). MicroRNAs: small RNAs with a big role in gene regulation. Nat Rev Genet.

[3] Zamore PD, Haley B (2005). Ribo-gnome: the big world of small RNAs. Science.

[4] Calin GA, Sevignani C, Dumitru CD, Hyslop T, Noch E, Yendamuri S, Shimizu M, Rattan S, Bullrich F, Negrini M, Croce CM (2004). Human microRNA genes are frequently located at fragile sites and genomic regions involved in cancers. Proc Natl Acad Sci U S A.

[5] Lu J, Getz G, Miska EA, Alvarez-Saavedra E, Lamb J, Peck D, Sweet-Cordero A, Ebert BL, Mak RH, Ferrando AA (2005). MicroRNA expression profiles classify human cancers. Nature.

[6] Garzon R, Fabbri M, Cimmino A, Calin GA, Croce CM (2006). MicroRNA expression and function in cancer. Trends Mol Med.

[7] Chou YT, Lin HH, Lien YC, Wang YH, Hong CF, Kao YR, Lin SC, Chang YC, Lin SY, Chen SJ (2010). EGFR promotes lung tumorigenesis by activating miR-7 through a Ras/ERK/Myc pathway that targets the Ets2 transcriptional repressor ERF. Cancer Res.

[8] Kong W, He L, Coppola M, Guo J, Esposito NN, Coppola D, Cheng JQ (2010). MicroRNA-155 regulates cell survival, growth, and chemosensitivity by targeting FOXO3a in breast cancer. J Biol Chem.

[9] Li N, Kaur S, Greshock J, Lassus H, Zhong X, Wang Y, Leminen A, Shao Z, Hu X, Liang S (2012). A combined array-based comparative genomic hybridization and functional library screening approach identifies mir-30d as an oncomir in cancer. Cancer Res.

[10] Schetter AJ, Leung SY, Sohn JJ, Zanetti KA, Bowman ED, Yanaihara N, Yuen ST, Chan TL, Kwong DL, Au GK (2008). MicroRNA expression profiles associated with prognosis and therapeutic outcome in colon adenocarcinoma. JAMA.

[11] Kefas B, Godlewski J, Comeau L, Li Y, Abounader R, Hawkinson M, Lee J, Fine H, Chiocca EA, Lawler S, Purow B (2008). microRNA-7 inhibits the epidermal growth factor receptor and the Akt pathway and is down-regulated in glioblastoma. Cancer Res.

[12] Godlewski J, Nowicki MO, Bronisz A, Williams S, Otsuki A, Nuovo G, Raychaudhury A, Newton HB, Chiocca EA, Lawler S (2008). Targeting of the Bmi-1 oncogene/stem cell renewal factor by microRNA-128 inhibits glioblastoma proliferation and self-renewal. Cancer Res.

[13] Chan JA, Krichevsky AM, Kosik KS (2005). MicroRNA-21 is an antiapoptotic factor in human glioblastoma cells. Cancer Res.

[14] Miele E, Buttarelli FR, Arcella A, Begalli F, Garg N, Silvano M, Po A, Baldi C, Carissimo G, Antonelli M (2014). High-throughput microRNA profiling of pediatric high-grade glioblastomas. Neuro-Oncology.

[15] Yang L, Shi CM, Chen L, Pang LX, Xu GF, Gu N, Zhu LJ, Guo XR, Ni YH, Ji CB (2015). The biological effects of hsa-miR-1908 in human adipocytes. Mol Biol Rep.

[16] Altomare DA, Testa JR (2005). Perturbations of the AKT signaling pathway in human cancer. Oncogene.

[17] Bellacosa A, Kumar CC, Di Cristofano A, Testa JR (2005). Activation of AKT kinases in cancer: implications for therapeutic targeting. Adv Cancer Res.

[18] Tsurutani J, Fukuoka J, Tsurutani H, Shih JH, Hewitt SM, Travis WD, Jen J, Dennis PA (2006). Evaluation of two phosphorylation sites improves the prognostic significance of Akt activation in non-small-cell lung cancer tumors. J Clin Oncol.

[19] Janmaat ML, Kruyt FA, Rodriguez JA, Giaccone G (2003). Response to epidermal growth factor receptor inhibitors in non-small cell lung cancer cells: limited antiproliferative effects and absence of apoptosis associated with persistent activity of extracellular signal-regulated kinase or Akt kinase pathways. Clin Cancer Res.

[20] Lee HY, Moon H, Chun KH, Chang YS, Hassan K, Ji L, Lotan R, Khuri FR, Hong WK (2004). Effects of insulin-like growth factor binding protein-3 and farnesyltransferase inhibitor SCH66336 on Akt expression and apoptosis in non-small-cell lung cancer cells. J Natl Cancer Inst.

[21] Potente M, Urbich C, Sasaki K, Hofmann WK, Heeschen C, Aicher A, Kollipara R, DePinho RA, Zeiher AM, Dimmeler S (2005). Involvement of Foxo transcription factors in angiogenesis and postnatal neovascularization. J Clin Invest.

[22] Dansen TB, Burgering BM (2008). Unravelling the tumor-suppressive functions of FOXO proteins. Trends Cell Biol.

[23] Fingar DC, Richardson CJ, Tee AR, Cheatham L, Tsou C, Blenis J (2004). mTOR controls cell cycle progression through its cell growth effectors S6K1 and 4E-BP1/eukaryotic translation initiation factor 4E. Mol Cell Biol.

[24] Alao JP (2007). The regulation of cyclin D1 degradation: roles in cancer development and the potential for therapeutic invention. Mol Cancer.

[25] Land SC, Tee AR (2007). Hypoxia-inducible factor 1alpha is regulated by the mammalian target of rapamycin (mTOR) via an mTOR signaling motif. J Biol Chem.

[26] Pisick E, Jagadeesh S, Salgia R (2004). Receptor tyrosine kinases and inhibitors in lung cancer. Sci World J.

[27] Blake DC, Mikse OR, Freeman WM, Herzog CR (2010). FOXO3a elicits a pro-apoptotic transcription program and cellular response to human lung carcinogen nicotine-derived nitrosaminoketone (NNK). Lung Cancer.

[28] Ambros V (2004). The functions of animal microRNAs. Nature.

[29] Calin GA, Croce CM (2006). MicroRNA-cancer connection: the beginning of a new tale. Cancer Res.

[30] Esquela-Kerscher A, Slack FJ (2006). Oncomirs - microRNAs with a role in cancer. Nat Rev Cancer.

[31] Li J, Yen C, Liaw D, Podsypanina K, Bose S, Wang SI, Puc J, Miliaresis C, Rodgers L, McCombie R (1997). PTEN, a putative protein tyrosine phosphatase gene mutated in human brain, breast, and prostate cancer. Science.

[32] Cully M, You H, Levine AJ, Mak TW (2006). Beyond PTEN mutations: the PI3K pathway as an integrator of multiple inputs during tumorigenesis. Nat Rev Cancer.

[33] Wang Y, Wang X, Zhang J, Sun G, Luo H, Kang C, Pu P, Jiang T, Liu N, You Y (2012). MicroRNAs involved in the EGFR/PTEN/AKT pathway in glioblastomas. J Neuro-Oncol.

[34] Liliental J, Moon SY, Lesche R, Mamillapalli R, Li D, Zheng Y, Sun H, Wu H (2000). Genetic deletion of the Pten tumor suppressor gene promotes cell motility by activation of Rac1 and Cdc42 GTPases. Curr Biol.

[35] Tamura M, Gu J, Matsumoto K, Aota S, Parsons R, Yamada KM (1998). Inhibition of cell migration, spreading, and focal adhesions by tumor suppressor PTEN. Science.

[36] Scagliotti GV, Selvaggi G, Novello S, Hirsch FR (2004). The biology of epidermal growth factor receptor in lung cancer. Clin Cancer Res.

[37] Zhang J, Han L, Zhang A, Wang Y, Yue X, You Y, Pu P, Kang C (2010). AKT2 expression is associated with glioblastoma malignant progression and required for cell survival and invasion. Oncol Rep.

[38] Jiang H, Shang X, Wu H, Gautam SC, Al-Holou S, Li C, Kuo J, Zhang L, Chopp M (2009). Resveratrol downregulates PI3K/Akt/mTOR signaling pathways in human U251 glioblastoma cells. J Exp Ther Oncol.

[39] Ruano Y, Mollejo M, Camacho FI, Rodriguez de Lope A, Fiano C, Ribalta T, Martinez P, Hernandez-Moneo JL, Melendez B (2008). Identification of survival-related genes of the phosphatidylinositol 3’-kinase signaling pathway in glioblastoma multiforme. Cancer.

[40] Smith JS, Tachibana I, Passe SM, Huntley BK, Borell TJ, Iturria N, O’Fallon JR, Schaefer PL, Scheithauer BW, James CD (2001). PTEN mutation, EGFR amplification, and outcome in patients with anaplastic astrocytoma and glioblastoma multiforme. J Natl Cancer Inst.

[41] Sasaki H, Zlatescu MC, Betensky RA, Ino Y, Cairncross JG, Louis DN (2001). PTEN is a target of chromosome 10q loss in anaplastic oligodendroglioblastomas and PTEN alterations are associated with poor prognosis. Am J Pathol.

[42] Ermoian RP, Furniss CS, Lamborn KR, Basila D, Berger MS, Gottschalk AR, Nicholas MK, Stokoe D, Haas-Kogan DA (2002). Dysregulation of PTEN and protein kinase B is associated with glioblastoma histology and patient survival. Clin Cancer Res.

[43] Raffel C, Frederick L, O’Fallon JR, Atherton-Skaff P, Perry A, Jenkins RB, James CD (1999). Analysis of oncogene and tumor suppressor gene alterations in pediatric malignant astrocytomas reveals reduced survival for patients with PTEN mutations. Clin Cancer Res.

[44] Thorarinsdottir HK, Santi M, McCarter R, Rushing EJ, Cornelison R, Jales A, MacDonald TJ (2008). Protein expression of platelet-derived growth factor receptor correlates with malignant histology and PTEN with survival in childhood glioblastomas. Clin Cancer Res.

[45] Li Y, Guessous F, Johnson EB, Eberhart CG, Li XN, Shu Q, Fan S, Lal B, Laterra J, Schiff D, Abounader R (2008). Functional and molecular interactions between the HGF/c-Met pathway and c-Myc in large-cell medulloblastoma. Lab Investig.

[46] Xia H, Ooi LL, Hui KM (2012). MiR-214 targets beta-catenin pathway to suppress invasion, stem-like traits and recurrence of human hepatocellular carcinoma. PLoS ONE.

