# Supplementary figures and images for: MicroRNA-1908 functions as a glioblastoma oncogene by suppressing PTEN tumor suppressor pathway
Source: Mol Cancer. 2015 Aug 12;14:154. doi: 10.1186/s12943-015-0423-0 (PMC4534015; doi:10.1186/s12943-015-0423-0)

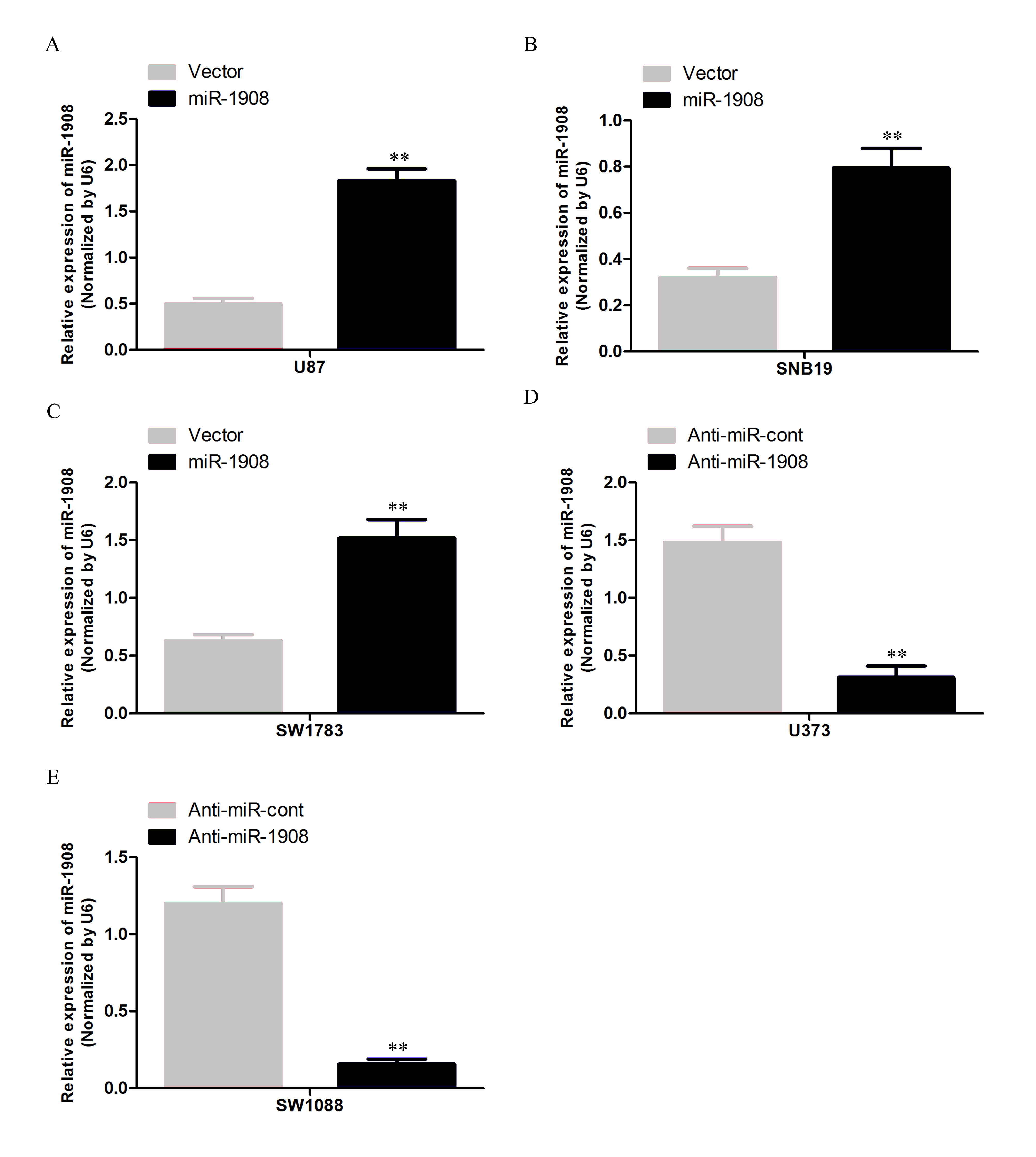

Supplement: Additional file 1: Figure S1. — Confirmation of miR-1908 expression in indicated cells. (A), (B), (C), (D) and (E) Quantification of miR-1908 in indicated cells. **P < 0.01. based on the Student t test. Error bars, SD. (JPEG 2379 kb) [file 12943_2015_423_MOESM1_ESM.jpg]

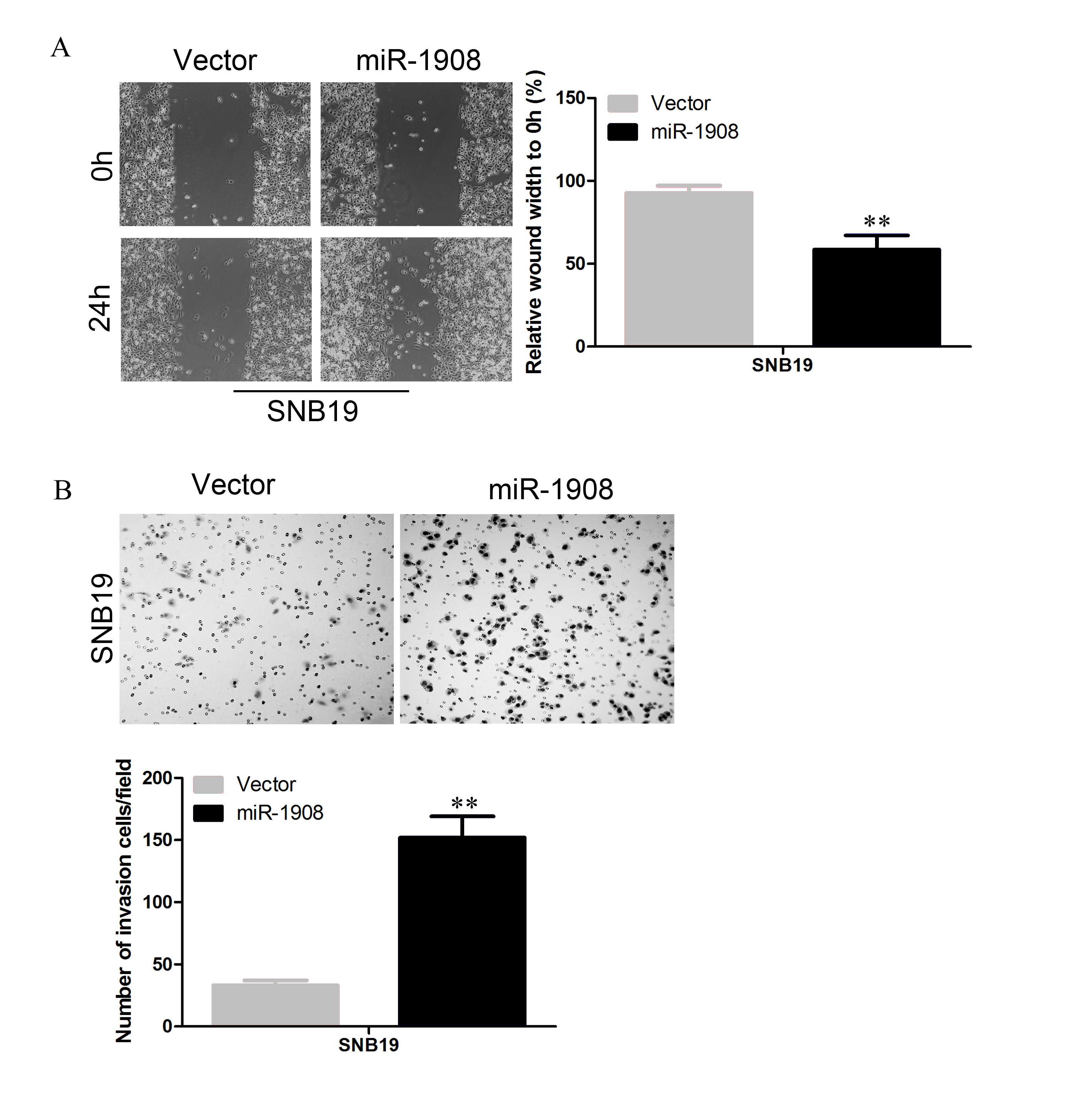

Supplement: Additional file 2: Figure S2. — miR-1908 promotes migration and invasion in SNB19 cells. (A) Scratch assay shows the effect of miR-1908 on migration in SNB19 cells. (B) Matrigel assay shows the effect of miR-1908 on invasion in SNB19 cells. **P < 0.01 based on the Student t test. Error bars, SD. (JPEG 2042 kb) [file 12943_2015_423_MOESM2_ESM.jpg]

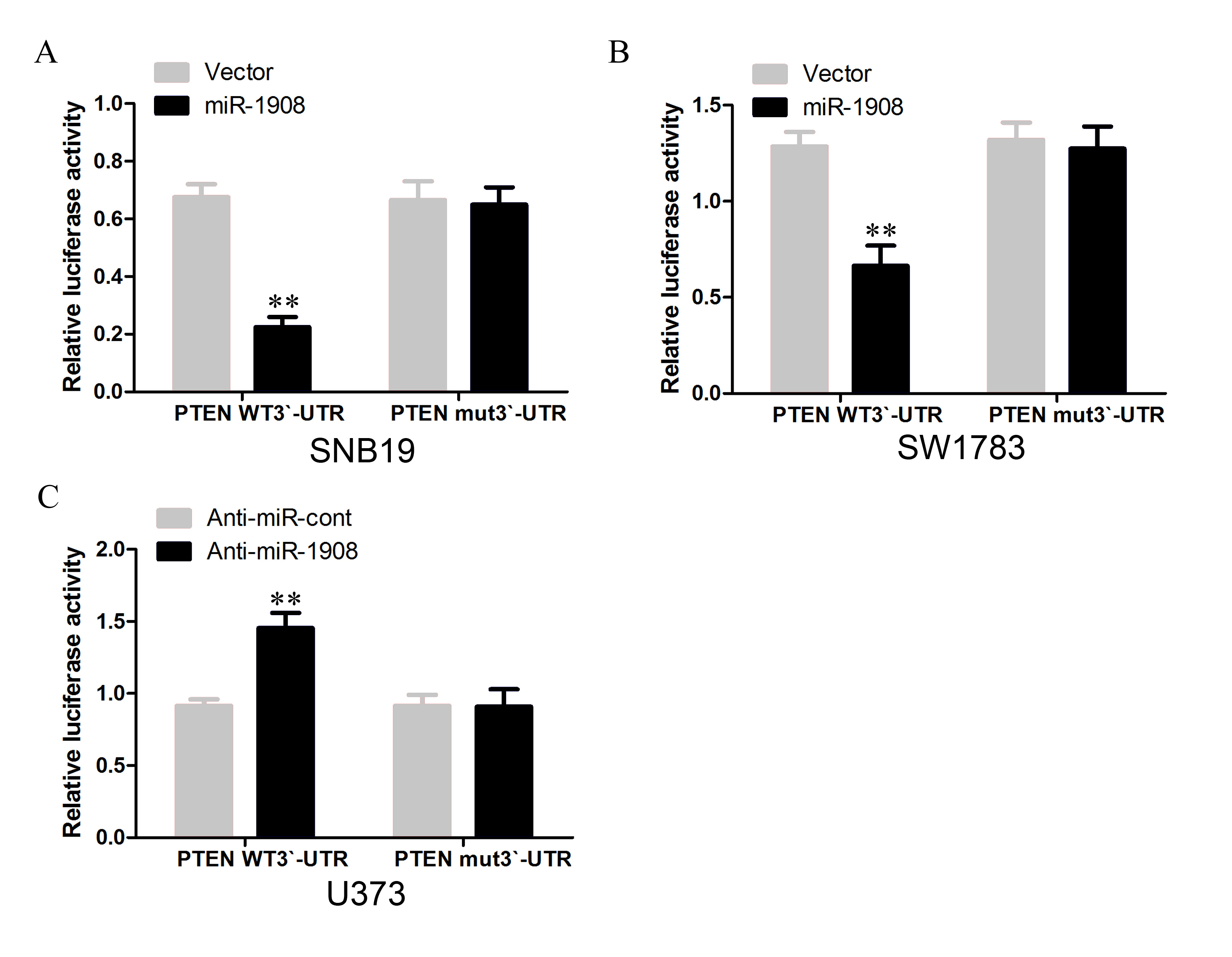

Supplement: Additional file 3: Figure S3. — miR-1908 inhibits PTEN activation in glioblastoma cells. (A), (B) and (C) Relative luciferase activity of PTEN in cells after co-transfection with wild type (Wt) or mutant (Mt) PTEN 3’-UTR reporter genes and miR-1908 mimics, anti-miR-1908 mimics or control. **P < 0.01 based on the Student t test. Error bars, SD. (JPEG 1115 kb) [file 12943_2015_423_MOESM3_ESM.jpg]

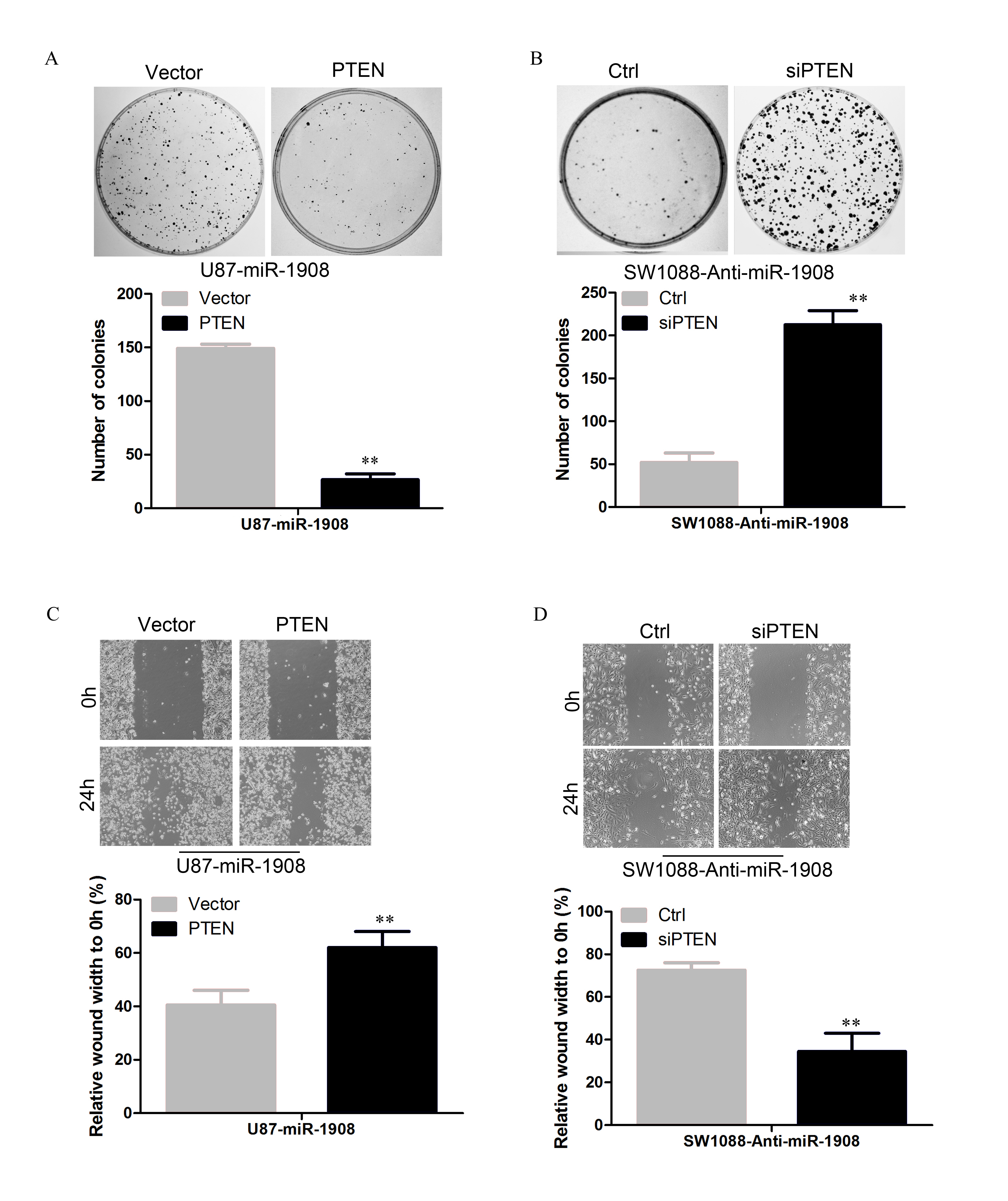

Supplement: Additional file 4: Figure S4. — Restoration of PTEN inverses miR-1908–induced proliferation and invasion in U87 or SW1088 cells. Colony formation (A) and scratch assay (C) show the effect on indicated cells after reexpression of PTEN. Colony formation (B) and scratch assay (D) show the effect on indicated cells after depletion of PTEN. **P < 0.01 based on the Student t test. Error bars, SD. (JPEG 4468 kb) [file 12943_2015_423_MOESM4_ESM.jpg]
